# Butyrate Produced by Commensal Bacteria Potentiates Phorbol Esters Induced AP-1 Response in Human Intestinal Epithelial Cells

**DOI:** 10.1371/journal.pone.0052869

**Published:** 2012-12-27

**Authors:** Malgorzata Nepelska, Antonietta Cultrone, Fabienne Béguet-Crespel, Karine Le Roux, Joël Doré, Vermulugesan Arulampalam, Hervé M. Blottière

**Affiliations:** 1 INRA, UMR 1319 MICALIS, Jouy-en-Josas, France; 2 AgroParisTech, UMR Micalis, Jouy-en-Josas, France; 3 Karolinska Institute, Department of Microbiology, Tumor and Cell Biology (MTC), Stockholm, Sweden; National Institute of Agronomic Research, France

## Abstract

The human intestine is a balanced ecosystem well suited for bacterial survival, colonization and growth, which has evolved to be beneficial both for the host and the commensal bacteria. Here, we investigated the effect of bacterial metabolites produced by commensal bacteria on AP-1 signaling pathway, which has a plethora of effects on host physiology. Using intestinal epithelial cell lines, HT-29 and Caco-2, stably transfected with AP-1-dependent luciferase reporter gene, we tested the effect of culture supernatant from 49 commensal strains. We observed that several bacteria were able to activate the AP-1 pathway and this was correlated to the amount of short chain fatty acids (SCFAs) produced. Besides being a major source of energy for epithelial cells, SCFAs have been shown to regulate several signaling pathways in these cells. We show that propionate and butyrate are potent activators of the AP-1 pathway, butyrate being the more efficient of the two. We also observed a strong synergistic activation of AP-1 pathway when using butyrate with PMA, a PKC activator. Moreover, butyrate enhanced the PMA-induced expression of c-fos and ERK1/2 phosphorylation, but not p38 and JNK. In conclusion, we showed that SCFAs especially butyrate regulate the AP-1 signaling pathway, a feature that may contribute to the physiological impact of the gut microbiota on the host. Our results provide support for the involvement of butyrate in modulating the action of PKC in colon cancer cells.

## Introduction

The gastrointestinal (GI) tract is a densely populated niche where finely tuned interactions occur between commensal microbiota and host cells. This creates a complex structure consisting of three closely interacting components: host, nutrition and microbiota. Commensal bacteria contribute to a wealth of GI functions, such as digestion of complex polysaccharides [1], production of essential nutrients or vitamins [2], barrier effect against pathogens, the maturation of the immune system [3], [4], regulation of host fat storage [5] and stimulation of intestinal angiogenesis. Accumulating data, suggest that bacterial metabolites and host transcription factors act as messengers in the crosstalk between these organisms [6], [7], [8], [9], [10], [11]. Short-chain fatty-acids (SCFA) are well-established components of this dialog. They are produced by commensal bacteria as byproducts of fiber fermentation, the principal ones being actetate, propionate, and butyrate [10], [11]. All SCFAs play an important role in the maintenance of a healthy colonic epithelium [12]. Butyrate the key SCFA produced by commensal bacteria, has been shown to modulate several signalling pathways in intestinal epithelial cells (IEC) including the activator protein-1 (AP-1) [11], [13]. Butyrate also exerts the most significant influence on IEC physiology [12] not only being the major source of energy but also acting as gene regulator in intestinal epithelial cells. AP-1 transcription factor is a dimeric complex whose major constituents belong to Jun and Fos protein subfamilies [14]. AP-1 plays important roles in cell proliferation, differentiation, transformation, cell migration, and apoptosis (for review, see [15], [16], [17]). The broad combinatorial possibilities provided by great numbers of AP-1 protein is mirrored in its binding specifcities and affinities and consequently spectrum of regulating genes [18]. The AP-1 binding site is located in promoter regions of many cytokines and chemokines such as IL-2, IL-3, IL-4, IL-6, IL-8, and tumor necrosis factor alpha (TNFα), [19], [20] as well as proteins controlling cell cycle, such as cyclin D1 [15]. The activity of individual AP-1 components can be regulated at various levels of transcription or through post-translational modifications and interactions with other proteins [16]. The members of the AP-1 family are phospho-proteins and their activity is affected by interactions with kinases and phosphatases [21]. Phosphorylation by the mitogen-activated protein kinases (ERK- and p38-MAPK, JNK) [22], Protein Kinase A and C (PKA, PKC), and glycogen synthase kinase-3 (GSK3) all affect AP-1 activity and function. Membrane GPCRs are known to transmit their effects, but intracellular signalling pathways need still to be fully elucidated (for review, see [23], [24]).

Butyrate acts as a differentiating agent [25] and activates PKC [26]. Interestingly, phorbol esters, similar to butyrate, exhibit differentiating potential involving activation of PKC [27]. Phorbol esters such a phorbol-12-myristate-13- acetate (PMA) are useful experimental analogs of diacylglycerol, the physiological activator of PKC [27] also exhibiting the potential to activate MAPK [28] and, as a consequence, the AP-1 response.

The AP-1 pathway is one of the most important for cell proliferation as well in intestinal epithelial differentiation [18]. The misbalance between these processes can be involved in human colorectal cancer [29].

The mechanisms behind AP-1 activating molecules in colonic epithelial cells have not been fully studied. One of the approaches to study the impact of metabolites emanating from commensal bacteria is through a combination of *in vitro* cultivation and cell reporter assays. This method allows the investigation of the molecular mechanisms of bacterial metabolites influencing host signalling pathways, and provides valuable insight on the impact of single bacterial metabolites. Therefore, in the current study, we investigated the impact of bacterial metabolites on IECs combining *in vitro* bacteria cultivation with functional assays of AP-1 response. Furthermore, we aimed to delineate the molecular mechanisms of the association of butyrate and PMA in the context of the AP-1 signal transduction pathway. Taking into account the vast range of processes influenced by butyrate and AP-1 signal transduction pathway as well as the fact that colonic epithelial cells are concurrently exposed to butyrate and factors activating AP-1 pathway, we investigated the molecular mechanism of this association.

## Materials and Methods

### Cell Culture and Reagents

The human epithelial cell lines HT-29 and Caco-2 were obtained from the American Type Culture Collection (ATCC, Rockville, MD). HT-29 cells were grown in RPMI 1640 and Caco-2 cells in DMEM. Both culture medium were supplemented with 2 mM L-glutamine, 50 IU/mL penicillin, 50 µg/mL streptomycin and 10% (or 20% for Caco_2) heat-inactivated fetal calf serum (FCS) in a humidified 5% CO2 atmosphere (or 10% for Caco-2) at 37°C. All culture media were from Lonza.

MAPK kinase inhibitors, U0126 (MEK1/2), PD98059 (MEK1), SP600125 (JNK) and SB203580 (P38); PKA inhibitor, H-89; and PKC inhibitor, Bisindolylmaleimide (BIM) were purchased from Calbiochem, deoxycholic acid (DCA), and ursodeoxycholic acid (UDCA)-from Sigma and used at 10 µM. Butyrate was used at 2 mM except in the dose-response study where a range of concentrations from 0.5 to 8 mM was used for all the short chain fatty acids (acetate, propionate, butyrate) and organic acids (lactate, succinate, and formate) tested. Trichostatin A (Cayman Chemical) was used at a 0.5 µM concentration; Epidermal Growth Factor (EGF) from Sigma was used at 10 ng/ml except for the dose response experiment; Phorbol myristate acetate (PMA) was from Sigma.

### Commensal Strains and Preparation of Conditioned Media

57 commensal strains were selected from the INRA Micalis collection and grown in anaerobic condition at 37°C using the Hungate culture method [30]. Screened strains and corresponding growth media are listed in [31].

At the end of the incubation period, OD and pH was measured and bacterial cultures were centrifuged at 5,000× g for 10 min. Bacteria conditioned media (CM) were then collected and filtered on 0.2 µm PES filters. Non-inoculated bacteria culture medium served as control. The organic acids were measured as previously described [32].

### Plasmid Construction and production of stable AP-1-luciferase reporter clones

Seven AP-1 repeated binding sites (TGACTAA), specific for monitoring PKC modulation, were cloned in the pcDNA3.1-Luciferase plasmid (referred as pAP-1-Luc). Stable HT-29 and Caco-2 reporter clones were obtained by transfecting cells with pAP1-Luc plasmid using the Nucleofector and appropriate kits (Amaxa, Lonza) following manufacturer's instructions. Cells underwent selection with Zeocin™ (Invitrogen; 50 µg/ml for HT29 and 100 µg/ml for Caco-2) and were cloned. Clones were selected for their response to PMA 0.1 µM after 24 h stimulation.

### Analyses of AP-1 activation: Luciferase Reporter Assay

For each experiment, reporter cells were seeded at 25,000 cells per well into 96-wells plates and incubated for 24 h. Then, cells were stimulated for 24 h with 10 µl of each tested bacteria CM in a final volume of 100 µl per well (i.e. 10% vol/vol). All conditions were tested in triplicate and at least three repetitions were performed. Luciferase activity, quantified as relative luminescence units (RLU), was measured using the ONE-Glo™ Luciferase Assay System (Promega) according to the manufacturer's instructions using a microplate reader (Infinite 200, Tecan).

### Real-Time PCR

Cell lines were seeded in 24 well culture plates using 0.5×10^6^ cells per well and cultured for 24 h before stimulation. After a stimulation time of 1 h and 6 h, total RNA was extracted using RNeasy mini-Kit (Qiagen). cDNA was synthesised from 1 µg of RNA using the High-Capacity cDNA Archive Kit (Applied Biosystems). qPCRs were carried out using an ABI Prism 7700 (Applied Biosystems) thermal cycler in a reaction volume of 20 µl. mRNA was quantified using SYBR Green (Applied Biosystems)-based quantitative real-time PCR for c-fos, cyclin D1 and β-actin used as normalization gene. The sample setups always included biological duplicates and experimental triplicates. Data are presented as fold change in the relative gene expression. Primers sequences: c-fos F-5′-CAGCGAGCAACTGAGAAGCC-3′, R - 5′-CGCTGTGAAGCAGAGCTGG-3′, cyclin D1 F-5′-TCCTCTCCAAAATGCCAGAG-3′ R -5′-TGAGGCGGTAGTAGGACAGG-3′, β-actin F-5′-CCTGGCACCCAGCACAAT-3′, R 5′-GCCGATCCACACGGAGTACT-3′.

### Western blot analysis

5×10^5^ HT-29 cells were seeded in 24 wells plates and starved for 24 h before stimulation, for various lengths of time, with 0.1 µM PMA, 2 mM butyrate, or association of both. Cells were washed twice with PBS and lysed in 2× Laemmli buffer. Proteins in samples were resolved in a denaturing 10% polyacrylamide gel and transferred to a nitrocellulose membrane (Amersham). Phosphorylation was detected using MAPK Family Antibody Sampler Kit (Cell signalling), followed by polyclonal rabbit horseradish peroxidase-coupled antibody (DAKO). The monoclonal anti-GAPDH-peroxidase (Sigma) was used as loading control. Finally, protein bands corresponding to the phosphorylated JNK, ERK1/2, p38 were revealed using the ECL™ detection system (Amersham Pharmacia Biotech) according to the manufacturers' instruction.

### Cell proliferation assays and measurements of alkaline phosphatase activity

Cell proliferation was measured after 24 h of incubation with PMA, Butyrate or its association using CellTiter 96® AQueous Non-Radioactive Cell Proliferation Assay (MTS) from Promega. Absorbance at 490 nm 30 min after the substrate was added to the cells. Alkaline phosphatase (AP) was measured in one-week post–confluent Caco-2 cells that were exposed for 48 h of treatment with PMA; butyrate or its association using Alkaline Phosphatase Assay Kit (Abcam; ab83369) according to the manufacturer's instructions.

### Statistical Analysis

Presented results are representative of at least 3 independent experiments. Results are expressed as mean ± SEM of representative triplicate measurements. Data were analyzed using Student's t test. PCA was done with ade4 R package [33]. Spearman correlations were done with the function cor.test of R (two-sided test).

## Results

### Bacterial metabolites are able to modulate AP-1 activity

**Figure 1:**
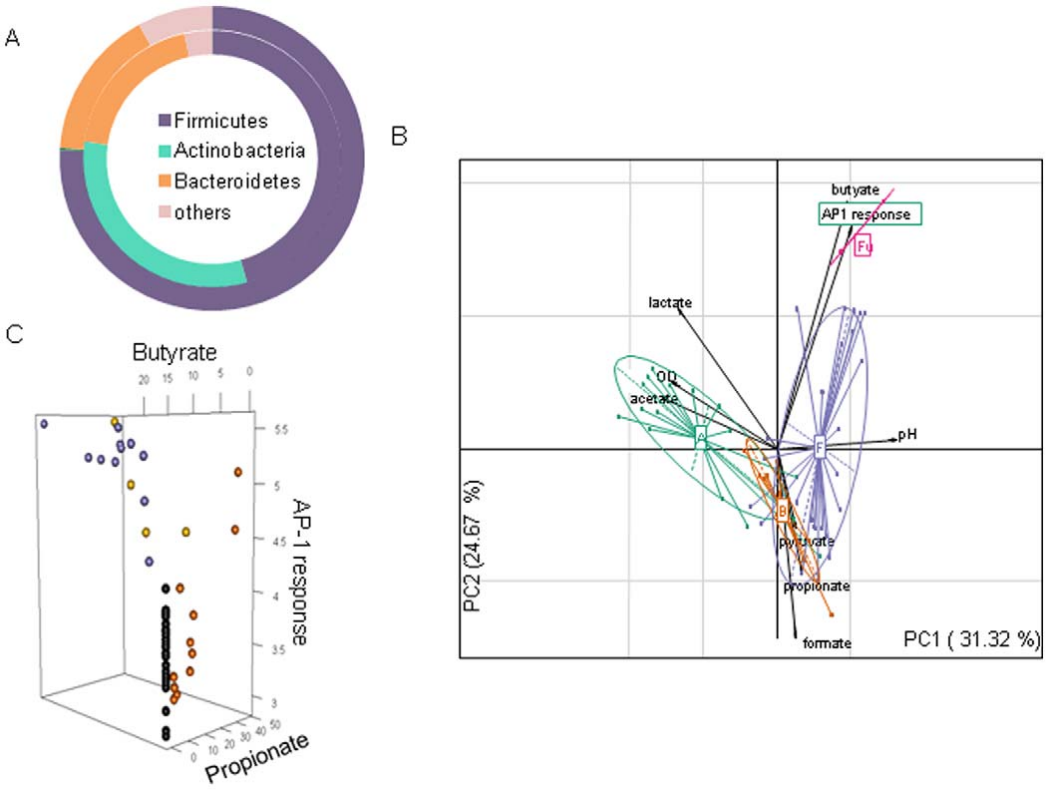
Correlation between bacterial metabolites production and AP1 activity. A) Bacterial phyla distribution in healthy human and in our selected bacterial collection. Inner part of the cycle represents the phyla distribution in our collection, outer part was based on the latest data from Tap et al. [60]. B) PCA showing the correlation between bacterial phyla, acid production and activation of AP-1 pathway. A positive correlation is observed for Firmicutes (violet) and Fusobacteria (pink). Bacteroidetes (orange), Actinobacteria (green). C) A detailed analysis using a three dimensional plot confirms the strong positive correlation between Butyrate (violet circles) producing bacteria and induction of AP-1 response and shows that no positive correlation is observed for propionate producing bacteria (orange circles) and non butyrate/propionate producing bacteria (black circles). Bacteria producing both butyrate and propionate are presented with yellow circles. AP-1 response (log RLU), propionate and butyrate concentration in (mM).

The culture supernatant of 49 selected bacteria were tested on HT-29/AP-1 reporter cell line showing that bacterial metabolites were able to differentially modulate the AP-1 pathway depending on the bacteria. Analogous results were obtained using another colonic reporter cell line Caco-2/AP-1 (data not shown). As presented on Figure 1A, these bacteria were selected to cover the 3 major phyla observed in the fecal metagenome, a third of the selected bacteria belongs to the core metagenome revealed from the MetaHIT study on 124 European individuals [34]. Although many bacterial phyla have been described as part of the intestinal microbiota, the microbial community is dominated by 3 major phyla, Bacteroidetes, Firmicutes, and Actinobacteria. Knowing that Actinobacteria (especially Bifidobacteria) have been shown to display beneficial effect on health, the tested collection was enriched in Bifidobacteria. Recent reports have associated Fusobacterium to colorectal cancer [35], [36], thus we also included several Fusobacteria. Proteobacteria were not represented in our collection because their interactions with the host have been widely studied elsewhere.

The major metabolites present in the CM have been determined using gas chromatography (for SCFA) and HPLC (for organic acid). Genes important for SCFA production are overrepresented and highly abundant in gut microbiota implicating the importance of carbohydrate fermentation as a key process in the colon. Through a principal component analysis (PCA), we tried to correlate the AP-1 transcriptional activity with the major metabolites secreted by commensal strains. Figure 1B reveals that the activation of AP-1 was strongly correlated with the presence of butyrate in the CM, but not with other SCFA or organic acid quantified. Indeed, a Pearson's product-moment correlation revealed a strongly significant correlation for butyrate (R = 0.780; p = 9.52e-13), a negative correlation was also noted for acetate (R = −0.316; p = 0.016). AP-1 activation was also associated with Firmicutes and Fusobacteria which are butyrate producers. Figure 1C presents the concentration dependent stimulation of the AP-1 pathway by commensal bacteria producing butyrate and propionate as well as the low modulatory capacity of the bacteria that do not produce these acids as an end product of fermentation. Propionate was capable of regulating AP-1 pathway only if present in high concentrations.

### Butyrate as the strongest activator of AP-1 among SCFA

**Figure 2:**
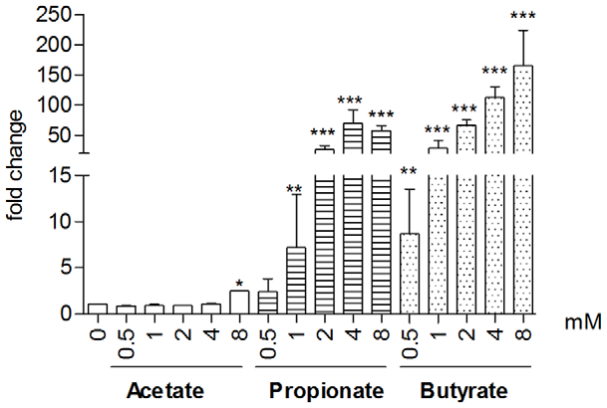
Dose-response of SCFA on AP-1 pathway activation. HT-29/AP-1 cells were exposed to increasing concentrations for 24 h. Data are mean ± standard error of the mean (SEM) of triplicate measurement of one representative of three independent experiments; ***P<0.001, **P<0.005, *P<0.05 as compared to control.

Subsequently, using HT-29/AP-1 reporter cells the observations made with CM were confirmed using increasing concentration of formate, acetate, propionate, butyrate, succinate and lactate. It is noteworthy that little amount of isobutyrate, valerate, and isovalerate were detected in the CM, never exceeding 2 mM, thus those SCFAs were not included in our study. Butyrate and to a lesser extent propionate strongly regulated AP-1 transcriptional activity in a dose dependent manner (Figure 2). Butyrate produced a significant activation at 0.5 mM (which correspond to a concentration in CM of 5 mM), whereas 1 mM (final concentration 10 mM) was needed to see activation with propionate with fold increases of 5.3 and 3.1, respectively. A very weak activation was observed with acetate when tested at 8 mM *i.e* a concentration in CM of 80 mM. The highest amount of acetate is produced by *B. adolescentis* and reached 76 mM. The other acid had no effect on AP-1 pathway whatever the concentration was (Figure S1). At 8 mM, lactate started to be toxic for HT-29 cells.

### Butyrate and PMA display synergistic activation on AP-1 pathway

**Figure 3:**
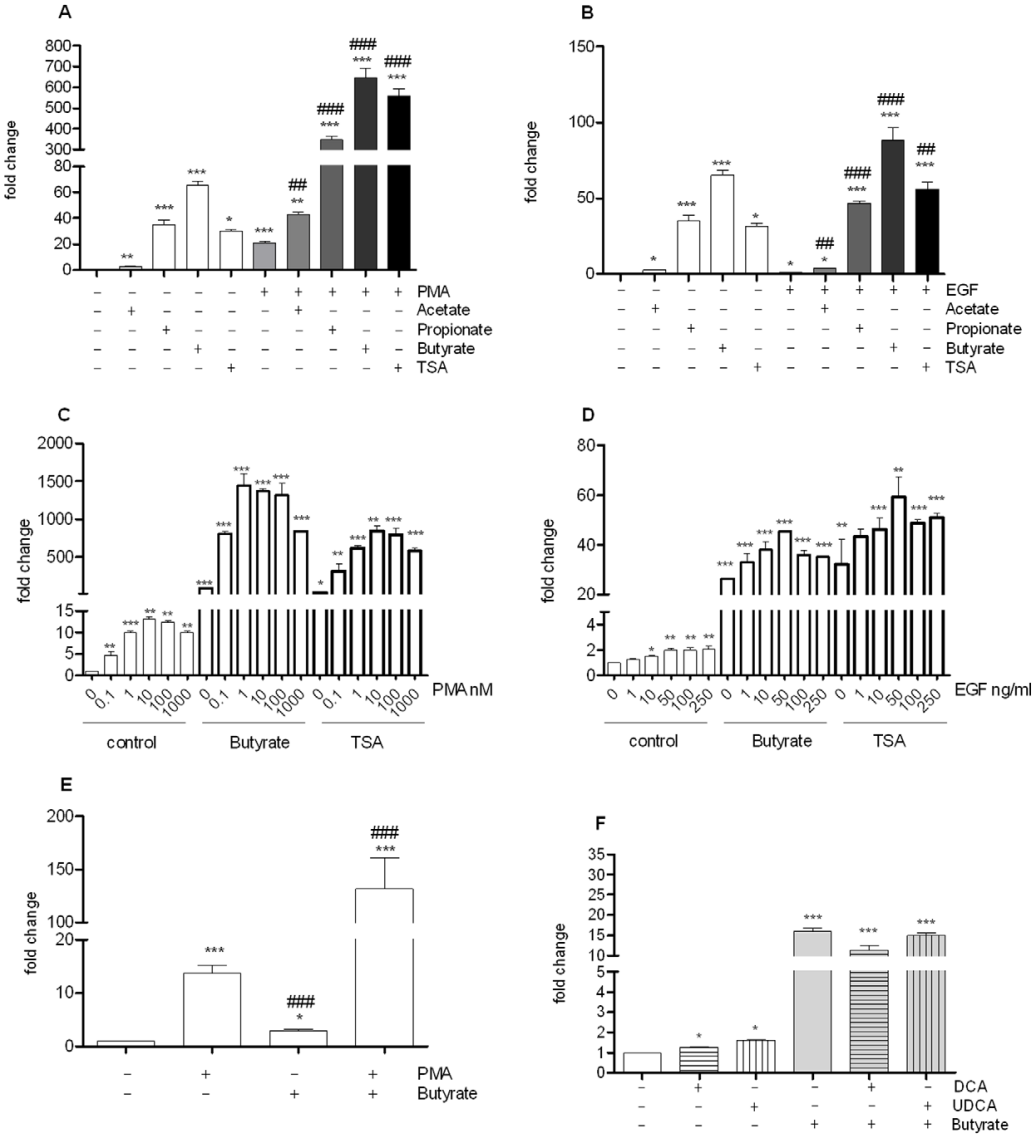
Activation of AP-1 pathway following HT-29 cell treatment with different SCFAs: acetate (8 mM), propionate (4 mM), butyrate (2 mM); or TSA (500 nM) and concomitantly treated with PMA (0.1 µM) (A) or EGF 10 ng/ml B). Dose response of PMA C) or EGF D) in the presence of butyrate (2 mM) or TSA (500 nM). Similar synergy upon association of PMA and butyrate is seen in another AP-1 reporter cell line Caco2/AP-1 E) Effect of bile acids: DCA (10 µM), UDCA (10 µM) associated or not with Butyrate (2 mM) (F). Data are mean ± standard error of the mean (SEM) of triplicate measurement of a representative of three independent experiments ***P<0.001, **P<0.005, *P<0.05 as compared with the control (Two way ANOVA); ### P<0.001, ## P<0.005, #P<0.05 as compared with the activator used (A, E : PMA ; B : EGF).

Butyrate and to a lesser extent propionate have been shown to activate different cell pathways through their effect as histone deacetylase (HDAC) inhibitors. We therefore tested trichostatin A (TSA), a well-characterized HDAC inhibitor, and observed that TSA activated the AP-1 pathway (Figure 3A). PMA, a standard AP-1 activator, was tested alone or in association with SCFAs. As expected, we observed that PMA activated the AP-1-luciferase reporter cells, however we observed a strong synergistic effect between SCFAs and PMA, resulting in 43, 340 and 659-fold increase for acetate, propionate and butyrate, respectively. A similar hyper-induction was noticed when PMA and TSA were associated indicating a link with HDAC inhibition.

We addressed the question whether this synergy was also true for another activator of the AP-1 pathway, EGF. However, when SCFAs were used together with EGF, only an additive effect was observed (Figure 3B). Dose response analysis confirmed the strong synergistic effect of butyrate or TSA and PMA (Figure 3C). The effect is seen for concentration of PMA as low as 0.1 nM and a plateau is reached with 0.1 µM. In contrast, an additive effect was noted when either butyrate or TSA was combined with EGF (Figure 3D).

The very strong synergistic effect is equally observed in another intestinal epithelial cell model, Caco-2/AP-1 (Figure 3E). Indeed, when butyrate and PMA were added together/simultaneously, AP-1 dependent luciferase activity increased by 131 fold. It is noteworthy that in Caco-2, PMA was a more potent AP-1 activator than butyrate. Since the synergistic effect is more pronounced in HT-29 cells, we decided to use them as a preferential model further study the crosstalk between butyrate and PMA on intestinal AP-1 pathway.

Finally, co-stimulation of cells with butyrate and secondary deconjugated bile acids deoxycholic acid (DCA), and ursodeoxycholic acid (UDCA) product of bacterial action on primary bile acids resulted in no or mild additive effects (Figure 3F).

### Butyrate and PMA exerted a synergistic effect on c-fos, but not on cyclin-D1

**Figure 4:**
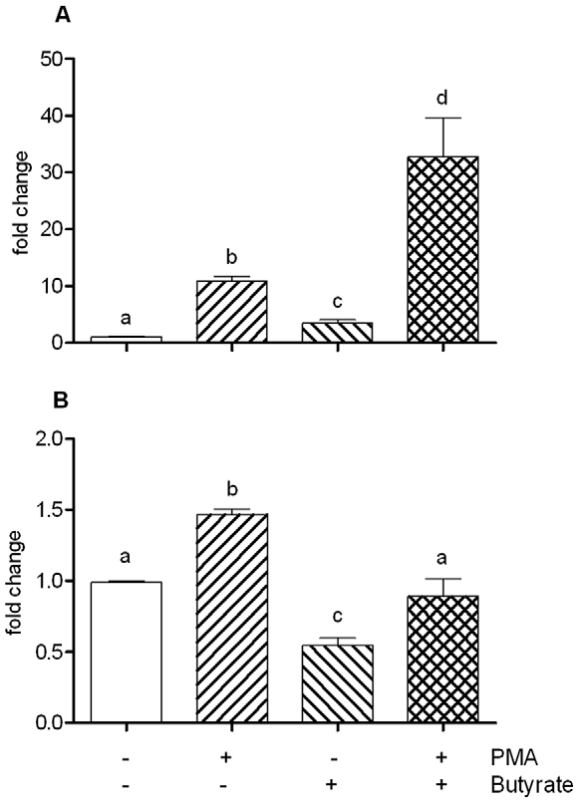
c-Fos A) and Cyclin D1 B) gene expression determined by Quantitative real-time PCR on total RNA extracted from cells exposed to PMA (0.1 µM), Na-Butyrate (2 mM), or both of them, for 1 h and 6 h, respectively. A positive, synergistic effect is observed on c-Fos expression for cells co-stimulated with PMA/Na-butyrate, while, in the same conditions, an opposite effect is observed for cycline D1. Data are mean + standard error of the mean (SEM) of (triplicate) measurement of a representative of three independent experiments. Different letters indicate statistically different results (p<0.05).

As the c-fos gene has previously been shown to be induced by PMA and by butyrate, we used its expression to further gauge AP-1 co-activation by PMA and butyrate. We confirmed that both compounds enhanced c-fos mRNA expression (Figure 4A), and furthermore, could synergistically affect its expression. Cyclin D1 expression however, although induced by PMA, is decreased by butyrate, and was not subject to co-activation (Figure 4B).

**Figure 5:**
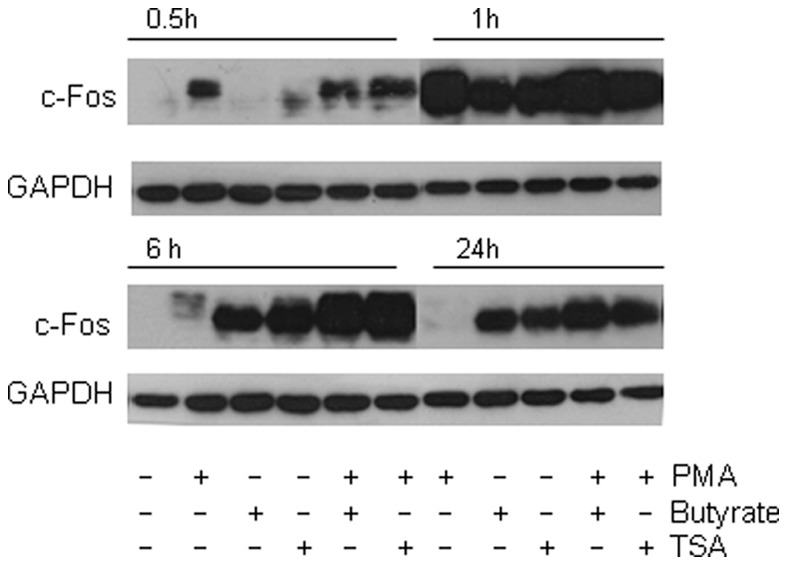
C-fos time course upon PMA induced activation. Cells were treated with PMA (0,1 µM), butyrate (2 mM), TSA 500 nM, or association of PMA with butyrate or TSA for 0,5, 1 h A), 6 and 24 h B) and total proteins were Western blotted for c-fos. A synergistic effect following PMA/Butyrate co-stimulation is observed after 6 h of stimulation and 24 h. Western blot of GAPDH (lower panel) was shown as loading control. Data are representative of three independent experiments.

The synergistic effect on c-fos was present at the protein level, but in a time dependent manner (Figure 5). Indeed, c-fos, which was barely detected without stimulation was induced by PMA alone 30 min. after stimulation, peaking at 1 h. Its expression decreased at 6 h and returned to baseline after 24 h. Conversely, the butyrate effect on c-fos expression was only detected at 1 h, albeit strongly. The effect was still observed at 6 and sustained at 24 h post stimulation. A similar pattern was observed with TSA, suggesting that co-activation was likely linked to the HDAC inhibitory properties of butyrate. The synergistic effect of PMA and butyrate (or TSA) is clearly seen after 6 h stimulation with both compounds, whereas at 30 min and 1 h it was only slightly increased when compared to PMA alone.

### Involvement of MAPkinases in PMA and Butyrate effect

**Figure 6:**
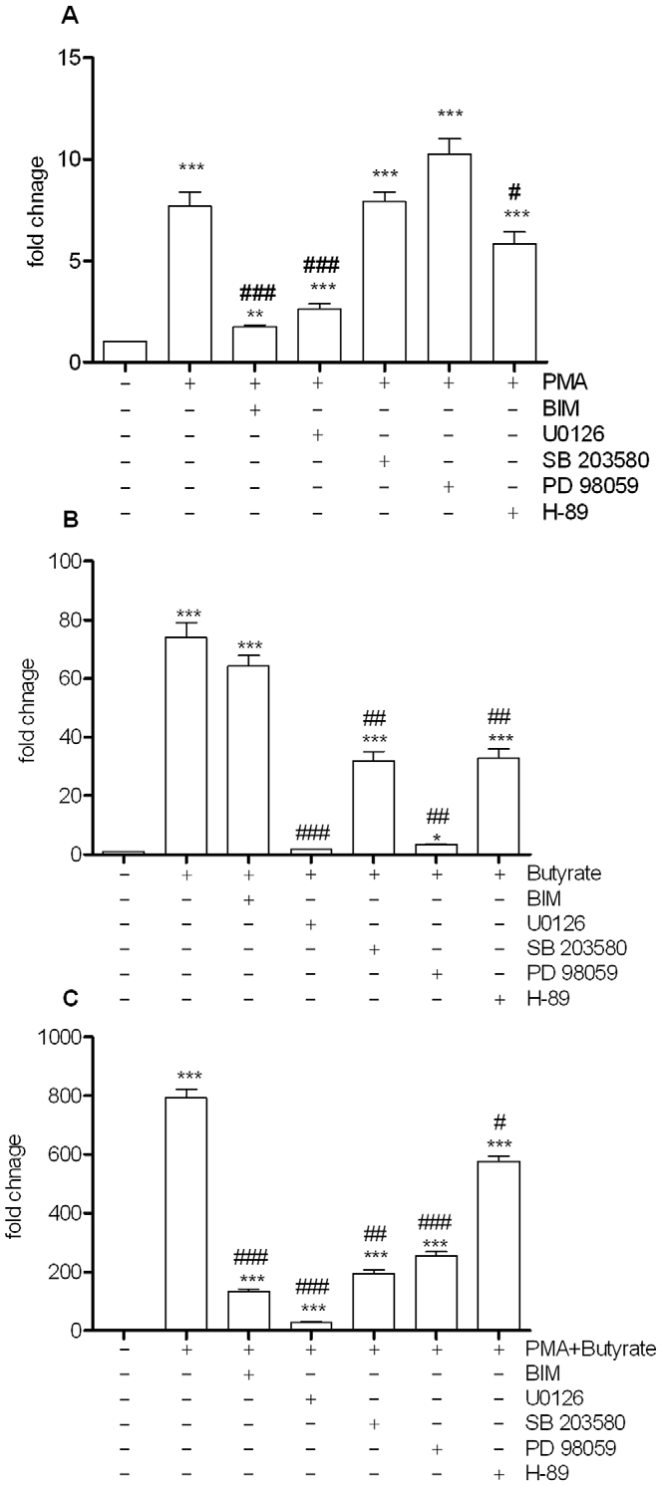
Effect of several kinase inhibitors on PMA (1 µM), Butyrate (2 mM) and PMA/Butyrate stimulated luciferase activity of HT-29 reporter cells. Bisindolylmaleimide (BIM 10 µM), UO126 (10 µM), SB203580 (10 µM), PD98059 (10 µM), H-89 (10 µM). Reporter gene activity was measured after 24 h stimulation. Results are mean + standard error of the mean (SEM) of triplicate measurements of a representative of three independent experiments ***P<0.001, **P<0.005, *P<0.05 compared with the control and ### P<0.001, ## P<0.005, #P<0.05. P values were determined by the t-test.

In an attempt to decipher the mechanisms involved in the AP-1 activation we used specific kinases inhibitors to map signal transduction pathways involved, The primary kinases involved in PMA inducible activity of AP-1 appear to be PKC and MEK/ERK, and to a lesser degree, PKA (Figure 6A, Figure S2). Activation using butyrate alone relied almost exclusively on MEK1/2, with no dependence on PKC (Figure 6B). A modicum of effect relied on p38 activation by butyrate.

The combination of PMA and butyrate (Figure 6C) resulted in a very strong synergistic activation of AP-1-dependent luciferase activity (789 fold increase of luciferase activity). With the exception of PKA, all other kinases studied here appeared necessary to potentiate this co-activation.

**Figure 7:**
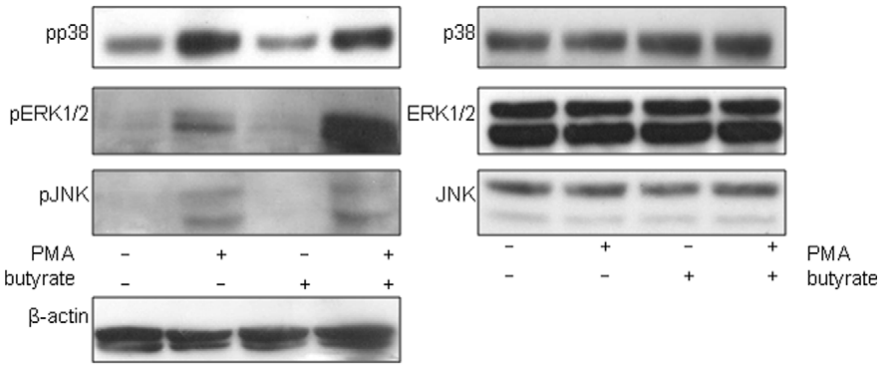
PMA induced activation of MAPK kinase pathway. Cells were treated with PMA (0.1 µM), butyrate (2 mM) or both of them for 30 min and total proteins were Western blotted for pp38, pERK1/2 and pJNK. A synergistic effect following PMA/Butyrate co-stimulation is observed in the case of ERK. Western blot of β-actin (lower panel) was shown as loading control. Data are representative of three independent experiments.

To further understand the signals through MAP kinase pathways required for the effects of butyrate and PMA, phosphorylation patterns of p38, ERK1/2 and JNK 3 kinases were assayed by Western blot (Figure 7). After 30 min PMA incubation with PMA, all 3 kinases were phosphorylated. In contrast, butyrate has a mild effect on ERK1/2 phosphorylation. The combination of PMA and butyrate however induced very strong phosphorylation of ERK1/2, confirming a key role for MEK/ERK pathway in this response. The synergistic effect observed on ERK1/2 but not on p38, nor on JNK was also observed in Caco-2 cells (data not shown).

### Effects of Butyrate, PMA and their association on cell proliferation and differentiation

**Figure 8:**
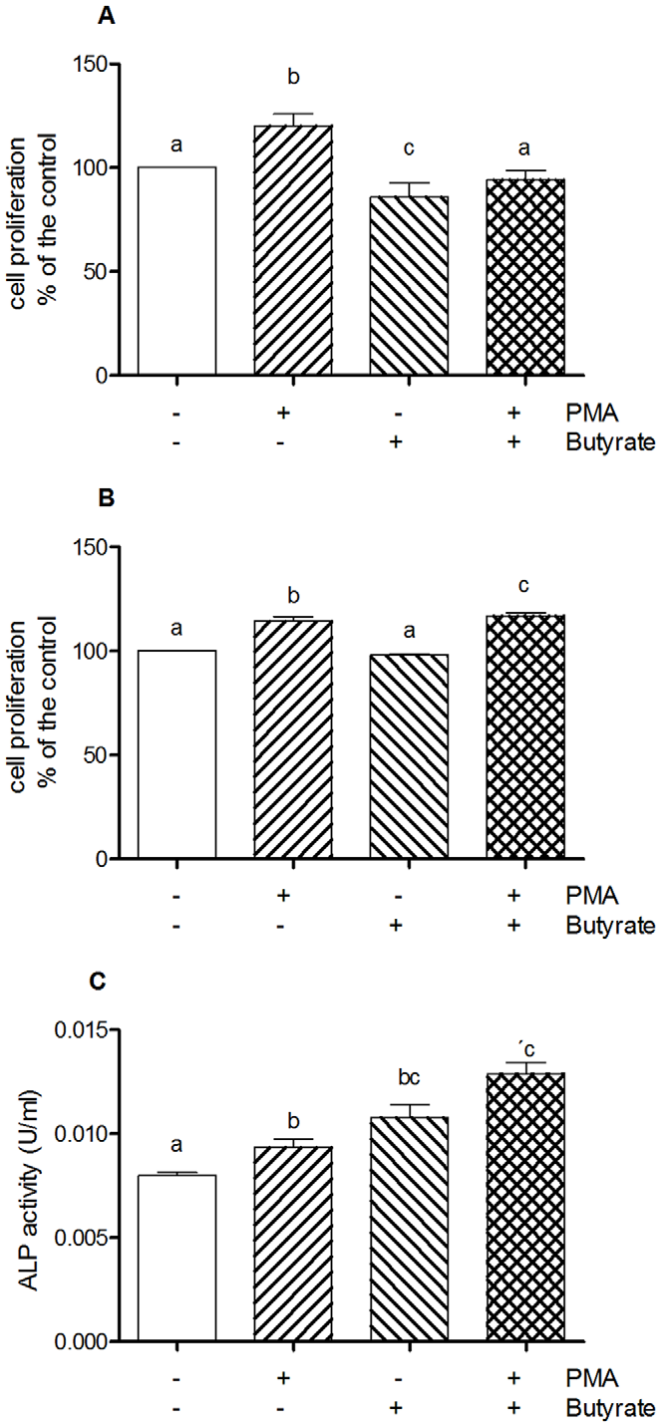
Effect of butyrate and on cell proliferation of Caco-2 A), HT-29 B) and on alkaline phosphatase activity as marker of differentiation in Caco-2 cells C). Data are representative of three independent experiments. Different letters indicate statistically different results (p<0.05).

Having deciphered the synergistic effect of butyrate and PMA on the AP1 pathway, we addressed the question of its potential impact on cell proliferation/death and differentiation. Exposure of HT-29 cells to Butyrate for 24 h resulted in a decrease in live cell number, whereas addition of PMA alone increased the number of cells (Figure 8B). Concomitant exposure of HT-29 cells to both butyrate and PMA resulted did not affect cell proliferation. Similar results were obtained with another colonic epithelial cell line Caco-2 Figure 8A. We have also investigated the role of Butyrate and PMA association on differentiation using Caco-2 cells (Figure 8C). Increased levels of alkaline phosphatase activity were observed upon addition of butyrate, PMA and both. The association of both resulted in an additive effect.

## Discussion

The healthy gut is densely populated with bacteria that greatly contribute to the physiology and well being of their human host. Their actions are mediated by the intimate contact, as well as the produced metabolites. With the present work, we established the impact of the bacterial metabolites on AP-1 pathway. Furthermore, we examined the effect of a concomitant administration of a PKC activator (PMA) and butyrate, which is mirrored in physiological condition in healthy colon.

Using the reporter cell lines, HT29/AP-1 and Caco-2/AP-1, that were designed for monitoring the activation of PKC and consequent AP-1 pathway modulation, we investigated the role of major bacterial metabolites (including SCFA) as well as important parameters like pH and OD (bacterial growth density) to challenge their role on AP-1 activation. Considering AP-1 activation by bacterial conditioned media, the major stimulatory effect was induced by CMs containing butyrate showing a very strong correlation (with p = 9.519e-13). Butyrate contained in CMs, and to the lesser extent propionate, was the driving force of AP-1 stimulation. However the culture media are complex mixture of metabolites and we cannot exclude that other molecules could also induce AP-1 response. The importance of butyrate was confirmed when the dose response to the different SCFAs were tested independently. Butyrate and propionate modulated significantly the AP-1 response. In an analogous experiment published from a previous study on NFkappaB modulation, no correlation with the stimulation of NFkappaB pathway by SCFAs alone was detected. Nevertheless, a strong correlation between NFkappaB stimulation and CMs containing propionate and butyrate was reported, which implied potential involvement of other bacterial metabolites driving the NFkappaB response [31].

The epithelial cells population is a dynamic continuum ranging from actively proliferating stem cells into terminally differentiated colonocytes. The colon is continuously flashed with molecules having the potential for promoting cell proliferation or differentiation. These factors are in constant dialog with key regulators of the response involving the host transcription factors. To maintain the balance between proliferation, differentiation, apoptosis but also immune responses, numerous regulatory mechanisms are required for the control of these processes.

There are many fundamental questions about how the gut microbiota can influence the homeostasis of the epithelium layer. Our results revealed that some bacterial metabolites (especially butyrate) are involved. However, since the luminal content is highly complex, we associated SCFAs with known PKC activators present in the human colon like diacylglycerol [37] and secondary bile acids [38]. We used butyrate and PMA to represent these 2 classes of molecules and further characterized the molecular mechanisms of this association.

Strong synergistic effects were observed upon addition of butyrate together with PMA, whereas propionate was less potent. Concomitant association of TSA with PMA also revealed this action. This finding may indicate an epigenetic mechanism of butyrate action indeed TSA is a well characterized histone deacetylase (HDAC) inhibitor [39]. Similarly, butyrate induces an accumulation of multiacetylated forms of histones [40], [41]. Histone acetylation alters the chromatin structure at the nucleosomal level, facilitating changes in DNA transcription. Moreover, HDAC are also involved in the control of the acetylation of lysine on key proteins including transcription factors. Interestingly it is a specific mechanism since the synergy is not found with all AP-1 activators tested, and that only key genes are regulated [42]. Nevertheless, the potential mechanism of HDAC/KDAC is above the aim of the present report.

This pronounced synergy was only seen with PMA neither with another AP-1 activator, EGF nor with the bile acids tested. EGF activation results in cellular proliferation, differentiation, and survival (for review, see [43]) and also utilise a Ras, MAPK pathway and PKC. This phenomena of synergistic action of PMA/butyrate was previously reported by Young et al where super-induction of TIMP-1 gene was shown whereas association of TGF-β1 to butyrate showed down regulation [44]. In line with this finding, we report that *c-fos*, a gene under the control of AP-1, showed similar pattern of super-induction, while it was not observed for cyclin D1. Butyrate was shown to attenuate cyclin D1 [41], [45] and decrease the level of other genes linked to cell proliferation such as *cdk1* as well as inducing differentiation-specific genes including *transglutaminase type I* [46]. Moreover, butyrate was shown to induce *c-fos* and *c-jun* in cancer cell lines [47]. The super induction of c-fos with parallel inhibition of cyclin D1 by PMA/butyrate underlines the role in the molecular regulatory mechanism of this association on genes regulating proliferation/differentiation. This goes in line with previously published data by Rickard et al who showed the synergistic effect of butyrate and PMA on maturation in human colonic epithelial cells using a pre-treatment with PMA before adding butyrate [48]. In our experiment, we did not observed such synergy on cell differentiation when butyrate and PMA were added simultaneously, but only an additive effect. As reported by Gaschott et al [49] combined exposure of HT-29 cells to both butyrate and 1,25-dihydroxy-Vitamin D3, extensively augmented HT-29 cell differentiation and reduced cell proliferation. 1,25-Dihydroxyvitamin D3 (1,25D) as PMA is a differentiating agent and have the capacity to induce AP-1 target genes [50]. It is noteworthy that concomitant exposure to butyrate and PMA resulted in a synergistic activation of the cytokine Thymic Stromal Lymphopoietin (TSLP) in HT-29 cells (Cultrone et al, personal communication), which may suggest a role in immunomodulation.

Trying to decipher details of the activation pathway, we investigated phosphorylation patterns of mitogen activated protein (MAP) kinase, as being upstream of AP-1 activation. The synergistic effect of PMA and butyrate was exclusively associated with phosphorylation of ERK1/2 but neither with JNK nor p38. ERK1/2 is primarily associated with cell growth, proliferation and differentiation whereas JNK and p38 are more connected to stress activated pathway. Consequently, super-induction of ERK1/2 contributed to increased activation of *c-fos*, gene that was proposed to serve as a “sensor” of duration of ERK1/2 activation [51].

As suggested by Chalmers et al, *c-fos* can serve as link between activation of ERK1/2 and AP-1 DNA binding action [52], indication that the biological outcome of ERK signalling is dependent upon the magnitude, duration and localization of its activation [53], [54]. Using specific array of MAP kinase inhibitors, we validated the observed effect. Inhibition of MEK1/2 by U0126 was able to block the stimulation in all the instances showing the key role of ERK 1/2 activation. The inhibition by U0126 was the strongest after activation by PMA or butyrate as well as on association of both. It is been shown that butyrate exhibit pro-apoptotic effects in cancer lines [55], [47] and consistent with these published findings, in our hands the p38 inhibitor was able to inhibit the effect of butyrate. Finally, PKA inhibitor was able to abolish the effect of all stimulation however the inhibition was ablated by approx 60% upon stimulation by butyrate. The involvement of PKA downstream of butyrate action was also reported previously [10]. Taken together these results indicate that wide ranges of butyrate dependent cellular effects are potentiated by parallel activation of by specific activator of PKC.

As a conclusion, in our study, we addressed in vitro the relevance of interaction between butyrate and other SCFAs with activators of PKC, such as diacylglycerols or deoxycholate, normally present in the lumen of the colon [56], [57]. Since concurrent exposure of cells to PMA and butyrate leads to synergistic effects on a key cellular process that is AP1 activation, the interaction of butyrate and PKC activators have potential to modulate the biology of the colon. Shift in diet profiles from fibers rich to Western diet is associated with higher risk of colon cancer. This increased dietary fat and cholesterol results in elevated secondary bile acids in the gut [58] additionally low SCFAs levels correlate negatively with health [59]. These issues highlight the possibility that this interaction may be an appropriate target for dietary and/or pharmacological intervention in the treatment or prevention of diseases of the large bowel. Also defining host signalling pathways regulated by the microbiota provides an opportunity to identify new therapeutic targets for promoting health.

## Supporting Information

Figure S1 **Dose-response of organic acid on AP-1 pathway activation.** HT-29/AP-1 cells were exposed to the increasing concentrations for 24 h. Data are mean ± standard error of the mean (SEM) of triplicate measurement of a representative of three independent experiments; ***P<0.001, **P<0.005, *P<0.05 as compared to control.
(TIF)

Figure S2 **Effect of several kinase inhibitors on the basal levels of the AP-1 response.** Bisindolylmaleimide (BIM 10 µM), UO126 (10 µM), SB203580 (10 µM), PD98059 (10 µM), H-89 (10 µM). Reporter gene activity was measured after 24 h stimulation. Results are mean + standard error of the mean (SEM) of triplicate measurements of a representative of three independent experiments.
(TIF)
