# Supplementary figures and images for: Butyrate Produced by Commensal Bacteria Potentiates Phorbol Esters Induced AP-1 Response in Human Intestinal Epithelial Cells
Source: PLoS One. 2012 Dec 27;7(12):e52869. doi: 10.1371/journal.pone.0052869 (PMC3531367; doi:10.1371/journal.pone.0052869)

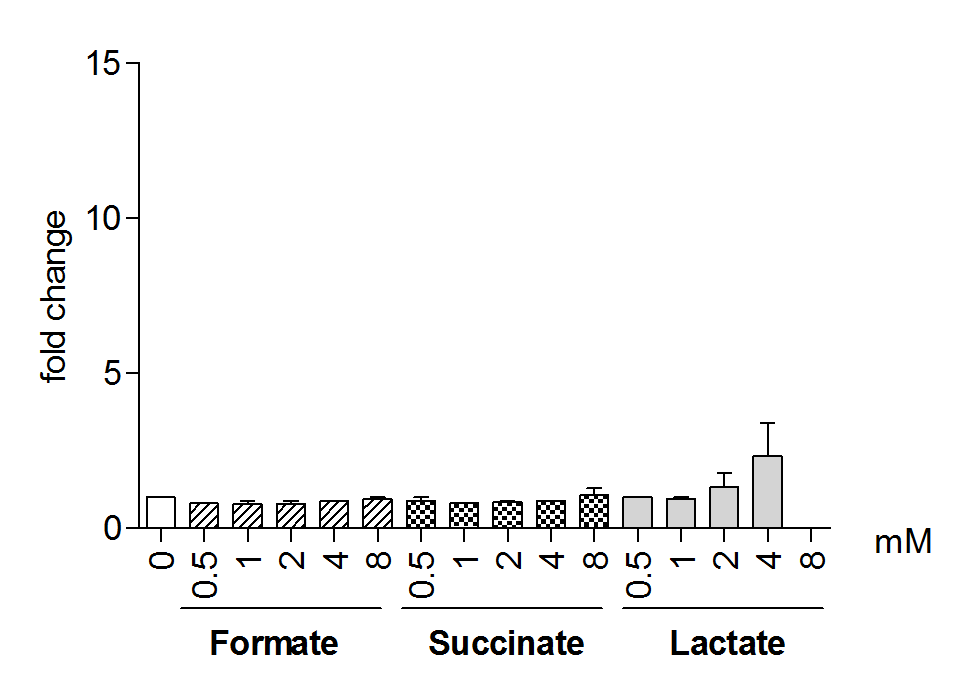

Supplement: Figure S1 — Dose-response of organic acid on AP-1 pathway activation. HT-29/AP-1 cells were exposed to the increasing concentrations for 24 h. Data are mean ± standard error of the mean (SEM) of triplicate measurement of a representative of three independent experiments; ***P<0.001, **P<0.005, *P<0.05 as compared to control. (TIF) [file pone.0052869.s001.tif]

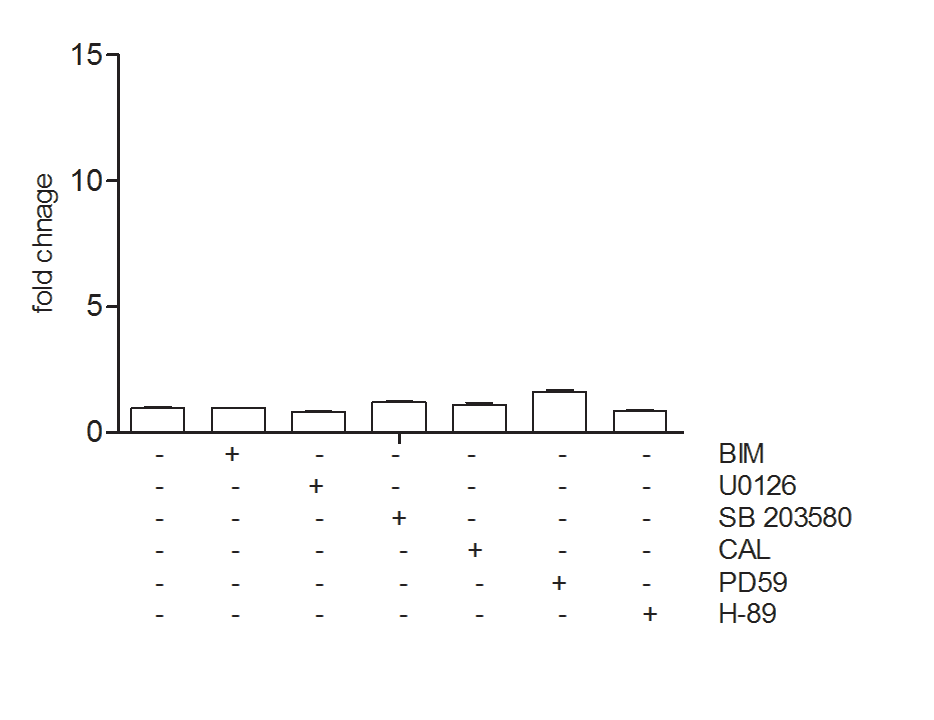

Supplement: Figure S2 — Effect of several kinase inhibitors on the basal levels of the AP-1 response. Bisindolylmaleimide (BIM 10 µM), UO126 (10 µM), SB203580 (10 µM), PD98059 (10 µM), H-89 (10 µM). Reporter gene activity was measured after 24 h stimulation. Results are mean + standard error of the mean (SEM) of triplicate measurements of a representative of three independent experiments. (TIF) [file pone.0052869.s002.tif]
